# Bridging the gap: Investigating somatologists’ knowledge of regulatory bodies in the Free State province of South Africa

**DOI:** 10.4102/hsag.v31i0.3371

**Published:** 2026-10-02

**Authors:** Adri Claassen, Juanita Jonker, Ronelle Jansen

**Affiliations:** 1 Department of Somatology, Faculty of Health and Environmental Sciences, Central University of Technology, Bloemfontein, South Africa; 2 School of Nursing, Faculty of Health Sciences, University of the Free State, Bloemfontein, South Africa

**Keywords:** somatology, regulatory knowledge, professional governance, statutory regulation, scope of practice, continuing professional development, professional associations, South Africa

## Abstract

**Background:**

Somatology practice in South Africa continues to expand through advanced aesthetic modalities; however, the profession remains without statutory regulation. Fragmented governance structures create risks regarding client safety, professional accountability and ethical practice. Understanding somatologists’ regulatory knowledge is important for future governance and regulatory progression.

**Aim:**

To investigate somatologists’ knowledge and understanding of regulatory entities relevant to the profession in the Free State province.

**Setting:**

The study included practising somatologists and salon owners in the Free State province of South Africa.

**Methods:**

A quantitative, descriptive cross-sectional design was used. Data were collected through a structured questionnaire distributed by two industry suppliers via WhatsApp and administered through QuestionPro. A total of 143 responses were included. Descriptive statistics assessed familiarity with relevant regulatory entities.

**Results:**

Respondents demonstrated strong recognition of voluntary entities such as the South African Association of Health and Skincare Professionals, but limited awareness of statutory councils. Considerable uncertainty existed regarding regulatory functions, registration requirements and organisational mandates. Knowledge of continuing professional development requirements was limited despite its high perceived value.

**Conclusion:**

The findings support future educational interventions, professional communication strategies and policy discussions regarding statutory regulation.

**Contribution:**

This study provides evidence on regulatory knowledge within an unregulated profession, informing curriculum development, policy discussions and future regulatory alignment.

## Introduction

The somatology profession in South Africa is undergoing substantial transformation as treatment scope and professional responsibilities continue to expand. Advanced modalities such as microneedling, chemical peels and laser-based therapies increasingly form part of routine practice, resulting in elevated clinical complexity and heightened expectations regarding somatologists’ competence (Ross, Borg & Garosi 2021; South African Association of Health and Skincare Professionals [SAAHSP] 2023). The absence of a statutory regulatory framework remains a persistent concern, as expanded practice is occurring without legally defined scopes, structured accountability mechanisms or enforceable standards safeguarding public well-being and ethical conduct (Louw & Esterhuizen 2021; Van Niekerk 2020).

These concerns become more evident when compared with international regulatory models. Across international contexts, aesthetic and wellness professions commonly operate under formalised regulatory bodies responsible for defining scopes of practice, developing standards and ensuring professional accountability (Cowie, Brönnum-Hansen & Sakellarides 2020). South African professional governance reflects a contrasting structure marked by voluntary associations and labour-directed bodies with limited regulatory authority (Cowie et al. 2020; National Bargaining Council for Hairdressing, Cosmetology, Beauty and Skincare Industry [HCSBC] 2022). Fragmentation within governance structures contributes to ongoing ambiguity regarding professional roles, ethical expectations and legal responsibility (Ross et al. 2021).

A deeper understanding of regulatory knowledge and the implications linked to professional governance is necessary to address the ambiguity within the industry. In the present paper, professionalisation theory provides the main conceptual perspective for interpreting regulatory knowledge, as the theory emphasises the development of formal governance structures, professional identity, standardised scopes of practice and regulatory accountability within emerging professions (Boulle & Louw 2021; Dambudzo 2015). Regulatory knowledge may therefore contribute to professional legitimacy, ethical alignment and preparedness for future integration into formal health governance systems.

This conceptual perspective aligns with regulatory governance literature, which emphasises coherent professional structures, clearly defined scopes of practice and formal accountability mechanisms (Boulle & Louw 2021; Dambudzo 2015). Such perspectives are relevant to somatology because expanding treatment technologies and rising public expectations require informed engagement with regulatory frameworks. Regulatory knowledge can strengthen ethical conduct, legal alignment and consistent professional judgement, while also supporting professional identity formation and public confidence in health-related services (Ross et al. 2021; SAAHSP 2023).

However, the regulatory system within the South African context presents a contrasting scenario. Professional governance in South Africa is shaped by multiple non-statutory structures that influence somatologists’ expectations, work environments and voluntary ethical guidelines. Voluntary associations, labour-oriented bodies and other organisational structures operate without unified statutory authority, resulting in inconsistency regarding accountability pathways and professional boundaries (Cowie et al. 2020; HCSBC 2022). Perspectives from health governance literature outline the influence of structural fragmentation on somatologists’ confidence, ethical alignment and engagement with governance processes (Louw & Esterhuizen 2021; Van der Merwe 2020; Vosloo 2009). Regulatory knowledge may support a more coherent interpretation of professional responsibilities across structurally diverse practice environments.

This structural complexity is intensified by technological advancements, and the consequent growth in advanced aesthetic modalities further emphasises the need for strengthened regulatory literacy. Increased utilisation of specialised technologies introduces greater clinical risk, elevated levels of responsibility and heightened ethical considerations for somatologists (Ross et al. 2021; SAAHSP 2023). Research relating to expanding aesthetic practice emphasises the value of informed knowledge of regulatory mandates, as regulatory knowledge supports safe treatment planning, ethical procedural implementation and alignment with public health governance priorities (Erasmus 2022; Louw & Esterhuizen 2021). After the present study had been concluded, the Department of Health issued a gazetted proposal exploring the possibility of placing somatology under the regulatory oversight of the Allied Health Professions Council of South Africa [AHPCSA], which further underscores the timeliness and relevance of the research study (South Africa. Department of Health 2025).

In addition to the regulatory developments, educational literature highlights the importance of preparing practitioners for governance requirements. In other words, educational literature reinforces the need to examine somatologists’ understanding of regulatory structures (SAAHSP 2023; South African Qualifications Authority [SAQA] 2023). Academic programmes require alignment with national governance priorities, ethical frameworks and evolving policy requirements (Department of Higher Education and Training [DHET] 2016; Pretorius 2018; SAQA 2022). Identification of knowledge gaps assists educators in refining curricula, strengthening preparation for professional practice and enhancing readiness for regulatory progression (Cowie et al. 2020; Louw & Esterhuizen 2021; Ross et al. 2021). Research focusing on regulatory knowledge in emerging health professions demonstrates the importance of structured education for promoting competent, legally aligned and ethically grounded service delivery (Mokoena & Govender 2019; Naidoo & Moodley 2022). Collectively, these educational considerations intersect with policy priorities, as regulatory knowledge informs curriculum development and readiness for statutory recognition (SAQA 2022; Van Niekerk 2020).

Policy-related relevance further strengthens the rationale for investigating regulatory knowledge. Insight into somatologists’ knowledge supports the evaluation of readiness for statutory recognition, the identification of communication gaps within governance structures and the assessment of alignment with broader national health system priorities (CHE 2020; HCSBC 2022; World Health Organization [WHO] 2021). Evidence generated through such investigation informs decision-making among policymakers, educators and professional associations committed to advancing professional accountability and public protection (DHET 2016; SAAHSP 2023).

Based on the literature, the present paper addresses the identified gap relating to regulatory knowledge among somatologists. Therefore, although the broader research forms part of an umbrella study entitled ‘Knowledge, Expectations and Practices of Somatologists in the Free State Regarding the Regulatory Bodies of the Somatology Profession in South Africa’, the current paper focuses exclusively on the knowledge component. The aim is to present and interpret findings regarding somatologists’ awareness and understanding of regulatory entities operating within the South African context, with particular attention to recognition of these entities, comprehension of their functional roles, and the influence of regulatory knowledge on professional engagement. The objectives of the paper are to evaluate knowledge of key governance structures, such as SAAHSP, the HCSBC, the AHPCSA, and the Health Professions Council of South Africa [HPCSA], and to highlight areas where understanding appears limited, as gaps in awareness may affect regulatory effectiveness, legal compliance, safe practice and ethical service delivery. Overall, such insight strengthens the evidence base required to support professional accountability and movement towards future regulatory alignment in the industry.

## Research methods and design

### Study design

The investigation was guided by a post-positivist paradigm that supports empirical observation, structured measurement, and critical reflection. The philosophical stance asserts the existence of an independent reality while also recognising the influence of researcher and participant bias during the research process (Park, Konge & Artino 2020; University of Nottingham 2019). Knowledge generation under a post-positivist orientation relies on rigorous methodology, validated instruments, and replicable procedures (Radford 2022). The paradigm aligned with the aim of identifying measurable patterns in somatologists’ regulatory knowledge, providing a foundation for evaluating levels of regulatory awareness and understanding (Bhandari 2021).

Within the selected paradigm, a quantitative, cross-sectional study design was adopted to direct the methodological approach (Creswell & Creswell 2018; McCombes 2021; McLeod 2019). The design enabled the collection of numerical data, supporting the identification of trends within the target population. A limited number of open-ended questions were included solely to provide contextual elaboration. The quantitative orientation remained dominant, and the descriptive responses served a supplementary interpretive function without altering the methodological foundation (Bryman 2016; Hanif 2023).

### Study population and sampling

The study population included qualified somatologists and salon owners operating within the Free State province. Both groups engage with regulatory structures within the somatology profession and were therefore relevant contributors to the investigation. Eligibility criteria required a 2- or 3-year somatology qualification or an equivalent credential, English literacy, and access to a mobile device with internet functionality. Individuals younger than 18 years, individuals without the required professional qualification, and individuals practising outside the Free State province were excluded.

Access to formal databases was restricted by data protection legislation. Therefore, a convenience sampling strategy was adopted (Bhandari 2021; McCombes 2021). Two established suppliers within the beauty industry assisted with participant recruitment. The two suppliers distributed invitations to respondents via WhatsApp, using existing client lists. A total of 569 invitations were distributed. Since no formal regulatory body for somatologists currently exists that could facilitate direct recruitment, and because the *Protection of Personal Information Act (POPIA)* restricts access to personal contact information, industry suppliers were utilised as recruitment gatekeepers. To minimise potential selection bias, the suppliers distributed the survey invitation to all eligible somatology clients within their databases rather than selectively inviting specific individuals. A total of 143 responses were received and included in the final dataset.

### Data collection

A structured questionnaire served as the primary data collection instrument developed to align with the research objectives (Radford 2022). The questionnaire included demographic variables and regulatory knowledge indicators. Knowledge questions primarily followed a five-point Likert-scale format (Bhandari 2021).

Content validity was supported through expert review by five specialists in somatology practice, somatology education and research methodology. Revisions strengthened clarity, wording and alignment with intended constructs. A pilot study involving five eligible respondents confirmed clarity, sequencing and completion time, and minor refinements were applied accordingly (Hanif 2023). Pilot respondents were recruited through the same suppliers but excluded from the main dataset.

The final questionnaire was administered via the QuestionPro platform (Austin, Texas, United States), which ensured encrypted data storage, anonymous participation and secure access. Two industry suppliers distributed invitations via WhatsApp, including an information letter, a consent form and a survey link. The questionnaire remained open for 3 months. To increase accessibility, printed versions were distributed by the same suppliers, returned upon completion and manually captured into the electronic database.

### Reliability and validity

Reliability reflected consistency in measurement and was strengthened through standardised wording, uniform Likert-scale formatting and controlled administration procedures (Bhandari 2021; Middleton 2023). All respondents completed the same standardised questionnaire, thereby supporting consistency in data collection.

Validity was addressed through expert review and pilot testing. The expert panel assessed clarity, relevance and alignment with the intended knowledge constructs, and refinements enhanced precision and conceptual coherence. Pilot feedback informed minor modifications to improve usability. In the present study, Cronbach’s alpha was not calculated to assess internal consistency, as the exploratory nature of the investigation prioritised the generation of contextual insights rather than scale development or psychometric evaluation. The knowledge items were designed as independent indicators assessing distinct aspects of regulatory knowledge rather than items within a single composite scale; consequently, internal consistency statistics such as Cronbach’s alpha were not considered appropriate. Reliability and validity were therefore established through expert review, pilot testing and alignment with the knowledge constructs, ensuring a credible representation of respondents’ regulatory knowledge.

### Data analysis

Electronic data and manually captured printed responses were exported to Microsoft Excel. Closed-ended questionnaire items generated numerical data, while limited open-ended items allowed respondents to elaborate on specific issues. Narrative inputs supported interpretation of numerical patterns without altering the quantitative emphasis of the investigation. A qualified biostatistician conducted descriptive statistical analysis using Statistical Package for the Social Sciences (SPSS) version 29 (IBM Corp., Armonk, New York, United States). Analysis included frequencies, percentages, means, and medians (McLeod 2019). The procedures aligned with the exploratory purpose of the investigation and supported the identification of patterns regarding knowledge of the respondents.

Narrative comments from the open-ended items were reviewed manually by the researcher. Recurring ideas were organised into descriptive categories. The qualitative component served a supplementary interpretive role and was not subjected to formal qualitative coding procedures. Quantitative and narrative components were interpreted separately to maintain methodological clarity (De Vos et al. 2011).

### Ethical considerations

Ethical clearance to conduct this study was obtained from the Health Sciences Research Ethics Committee (HSREC), Faculty of Health Sciences, University of the Free State (No. UFS-HSD2024/0676/1806). Ethical clearance was obtained prior to conducting the study. Participation was voluntary, and informed consent was required before accessing the questionnaire. No incentives were offered to participants.

Electronic data were stored on encrypted servers with password protection. Printed questionnaires were stored in a locked cabinet and destroyed after digitisation. Access to all data was restricted to the researcher, supervisors, and the biostatistician. Confidentiality was upheld throughout the research process (Saunders, Lewis & Thornhill 2003).

## Results

The results summarise the demographic characteristics of respondents and outline significant patterns relating to regulatory knowledge, familiarity with governance structures, membership behaviour, guideline awareness and continuing professional development.

Sample sizes vary across analyses because respondents could choose not to answer individual questionnaire items; percentages are therefore reported using the number of valid responses for each item. A total number of 143 questionnaire responses were obtained. The demographic profile indicates a predominantly early- to mid-career respondent group, with most respondents falling between 25 and 44 years (Table 1). Industry experience ranged from newly qualified somatologists to practitioners with more than 26 years of professional experience. Most respondents were actively employed in the somatology industry, and their professional roles were mainly client-facing, particularly in salon ownership and therapist positions. Overall, the respondent profile provides a relevant practice-based context for interpreting the regulatory knowledge findings presented below.

**TABLE 1:** Demographic profile of respondents.

Variable	Category	%
Age (years) (*n* = 125)	18–24	24.0
	25–34	31.0
	35–44	27.0
	45–54	11.0
	55+	6.0
Highest qualification (*n* = 115)	Certificate (1 year)	4.3
	Certificate (2–3 years)	6.1
	Diploma in Somatology (2–3 years)	58.3
	Advanced Diploma (1 year)	9.6
	Postgraduate Diploma	4.3
	Bachelor of Health Sciences	11.3
	Master’s degree	2.6
	Other	3.5
Industry experience (years) (*n* = 114)	1–5	15.8
	6–10	28.1
	11–15	12.3
	16–20	13.2
	21–25	11.4
	26+	19.3
Employment status (*n* = 112)	Employed in somatology	75.0
	Not employed	20.5
	Other	4.5
Primary professional role (*n* = 82)	Employer or salon owner	43.5
	Therapist	36.5
	Independent contractor	9.4
	Manager	3.5
	Lecturer	3.5

Note: Percentages are based on valid responses for each demographic variable. The number of valid responses varied because not all respondents completed every questionnaire item.

The following results describe respondents’ regulatory knowledge, including knowledge of regulatory entities within somatology, understanding of their functions and familiarity with professional standards guiding practice.

### Regulatory knowledge

This section presents respondents’ knowledge of regulatory structures relevant to the somatology profession. Specifically, it examines recognition and familiarity with regulatory entities, understanding of their functions and responsibilities, membership patterns, knowledge of regulatory guidelines and registration requirements, and awareness of continuing professional development (CPD) requirements.

#### Knowledge of regulatory structures

As shown in Figure 1, 71% (71/100) of respondents reported knowledge of regulatory entities (*n* = 100). Knowledge of professional standards was based on 118 valid responses (*n* = 118), with just over half reporting awareness of standards guiding professional conduct.

**FIGURE 1:**
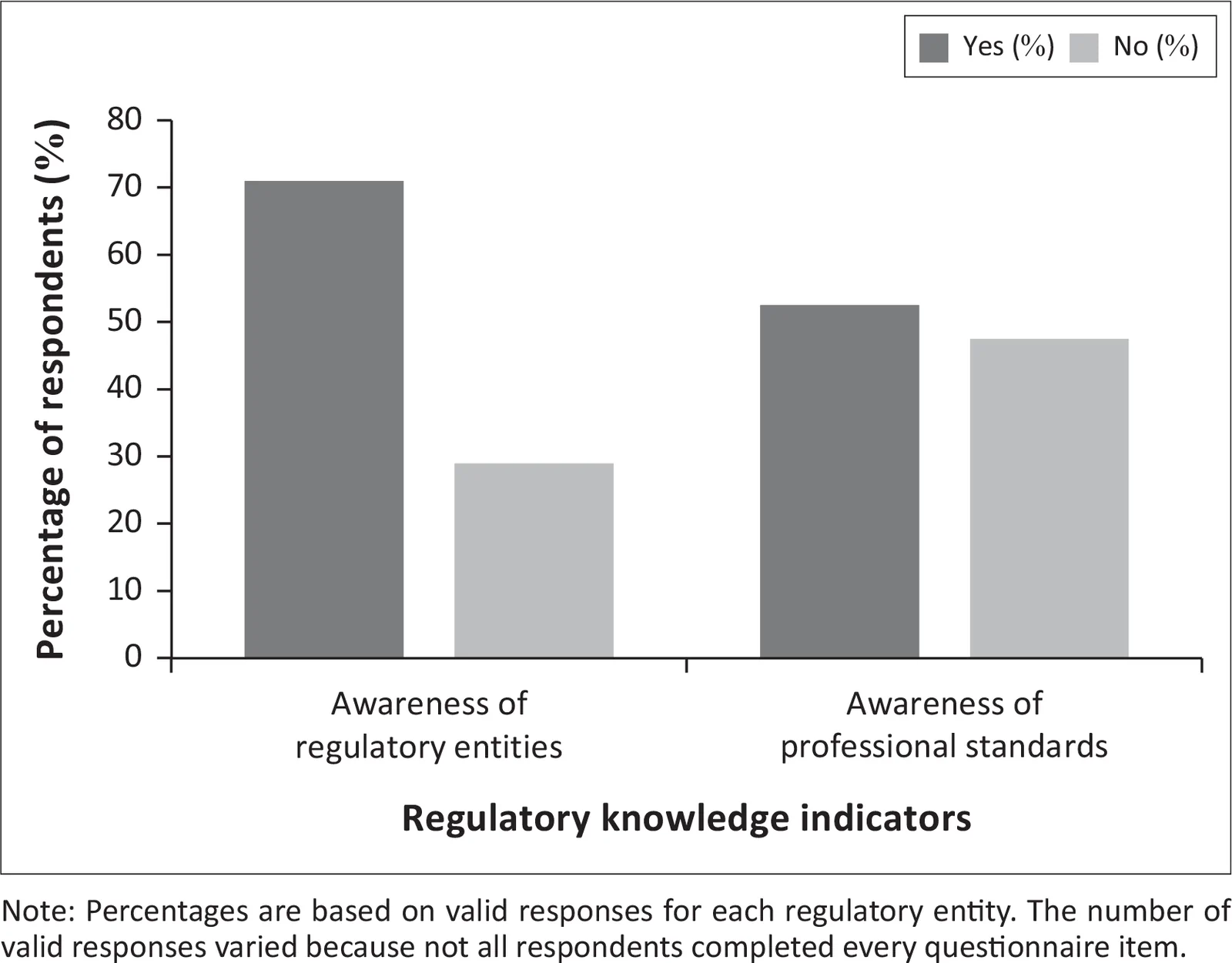
Knowledge of regulatory structures (Regulatory entities: *n* = 100; Professional standards: *n* = 118).

#### Recognition and familiarity

Table 2 presents respondents’ knowledge of the primary regulatory entities. South African Association of Health and Skincare Professionals was the entity most frequently recognised as the primary regulatory body (85.2%), followed by HCSBC (42.9%). Recognition was considerably lower for the Beauty Health and Skincare Employers Association (BHFSA), the HPCSA, the Employers’ Organisation for Hairdressing, Cosmetology and Beauty (EOHCB) and the AHPCSA, ranging from 26.5% to 33.3%. Uncertainty was greatest for BHFSA (42.5%), AHPCSA (36.1%) and EOHCB (34.1%).

**TABLE 2:** Knowledge of primary entities and familiarity levels.

Entity	%			Highest familiarity response	%
	Primary	Not primary	Uncertain		
AHPCSA	26.5	18.1	36.1	Slightly familiar	47.4
BHFSA	32.5	23.8	42.5	Not familiar	55.3
EOHCB	30.5	28.0	34.1	Not familiar	53.9
HCSBC	42.9	23.8	27.4	Not familiar	46.1
HPCSA	33.3	25.6	29.5	Slightly familiar	31.6
SAAHSP	85.2	3.7	9.9	Extremely familiar	31.1

Note: Percentages are based on valid responses for each regulatory entity. The number of valid responses varied because not all respondents completed every questionnaire item.

HCSBC, National Bargaining Council for Hairdressing, Cosmetology, Beauty and Skincare Industry; AHPCSA, Allied Health Professions Council of South Africa; HPCSA, Health Professions Council of South Africa; BHFSA, Beauty Health and Skincare Employers Association; EOHCB, Employers’ Organisation for Hairdressing, Cosmetology and Beauty; SAAHSP, South African Association of Health and Skincare Professionals.

Familiarity with regulatory entities reflected a similar pattern. South African Association of Health and Skincare Professionals again reflected the strongest response, recording the highest level of extreme familiarity at 31.1%. In contrast, unfamiliarity was more apparent for several other entities. More than half of respondents reported unfamiliarity with BHFSA, EOHCB and HCSBC (see Table 2).

#### Regulatory oversight responsibilities and functional roles

Table 3 summarises respondents’ knowledge of regulatory oversight responsibilities. South African Association of Health and Skincare Professionals received the highest level of oversight recognition (35.5%). Other entities reflected lower levels of oversight attribution, including HCSBC, EOHCB, BHFSA, AHPCSA and HPCSA.

**TABLE 3:** Knowledge of regulatory oversight and functional roles.

Entity	%			Most selected functional role	%
	Oversight yes	Oversight no	Uncertain		
AHPCSA	15.2	11.6	19.6	Statutory health body	36.4
BHFSA	15.2	13.0	18.8	Workplace representative	24.1
EOHCB	15.9	13.8	15.9	Workplace representative	28.3
HCSBC	18.1	13.8	13.8	Workplace representative	17.0
HPCSA	13.8	21.0	10.1	Statutory health body	34.0
SAAHSP	35.5	3.6	6.5	Statutory + training aligned	39.6 each

Note: Percentages are based on valid responses for each regulatory entity. The number of valid responses varied because not all respondents completed every questionnaire item.

HCSBC, National Bargaining Council for Hairdressing, Cosmetology, Beauty and Skincare Industry; AHPCSA, Allied Health Professions Council of South Africa; HPCSA, Health Professions Council of South Africa; BHFSA, Beauty Health and Skincare Employers Association; EOHCB, Employers’ Organisation for Hairdressing, Cosmetology and Beauty; SAAHSP, South African Association of Health and Skincare Professionals.

Table 3 also presents respondents’ understanding of the functional roles of the regulatory entities. AHPCSA and HPCSA were most frequently associated with statutory health functions. Workplace-related representation was most commonly attributed to BHFSA, EOHCB and HCSBC. South African Association of Health and Skincare Professionals reflected its strongest association with statutory and training-related functions.

#### Membership patterns and challenges influencing affiliation

Figure 2 illustrates membership patterns among respondents (*n* = 52). South African Association of Health and Skincare Professionals had the highest membership, whereas the remaining entities showed minimal affiliation.

**FIGURE 2:**
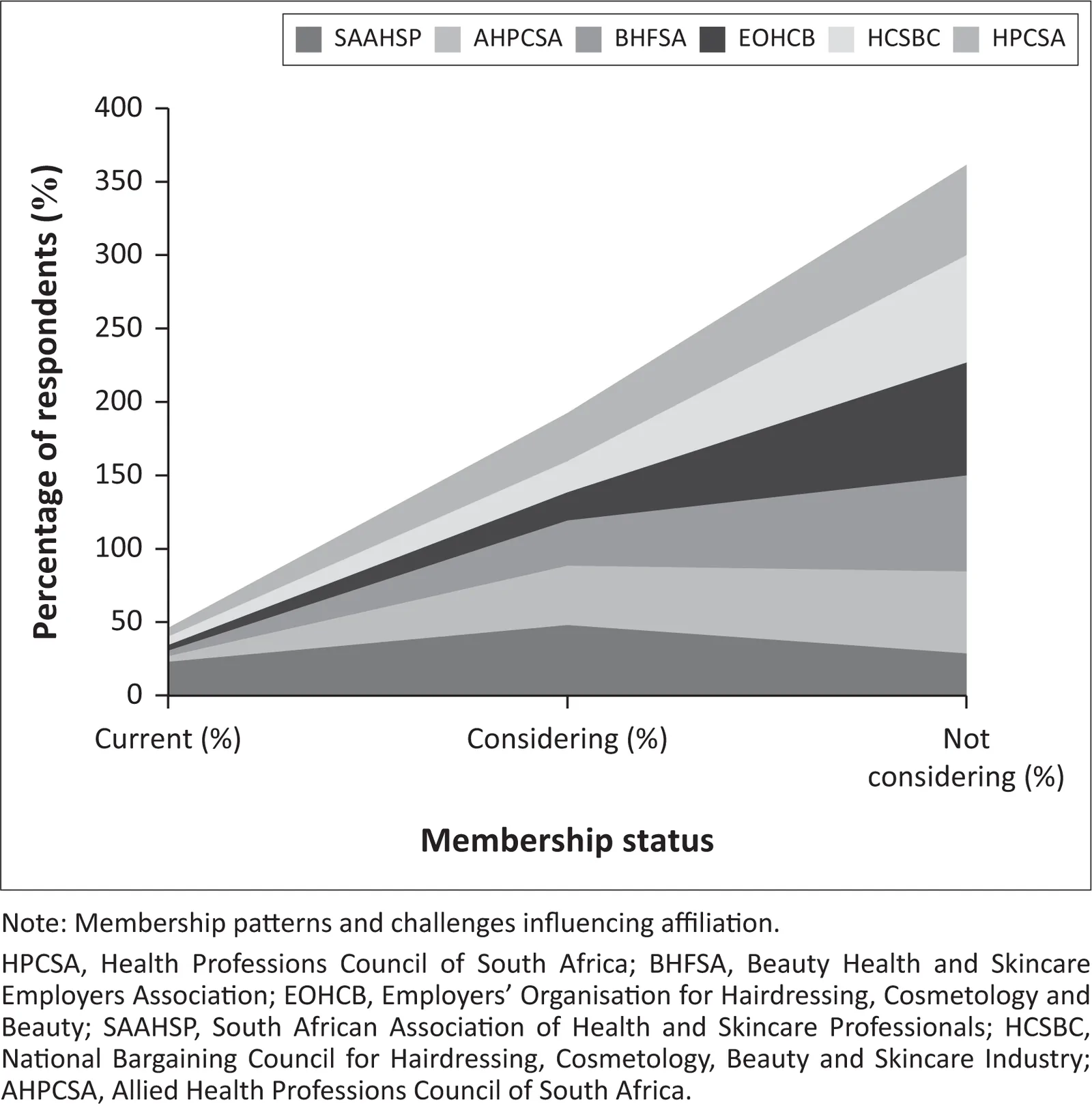
Membership patterns and future membership intentions (*n* = 52).

Figure 2 also illustrates respondents’ interest in future membership. Interest in future affiliation ranged from 19.2% for EOHCB to 48.1% for SAAHSP, whereas non-membership ranged from 28.8% to 76.9% across the entities.

Figure 3 presents the challenges influencing affiliation (*n* = 51). Uncertainty regarding which entity to join was the most frequently reported challenge (54.9%), followed by financial constraints and limited knowledge of membership benefits (41.2% each).

**FIGURE 3:**
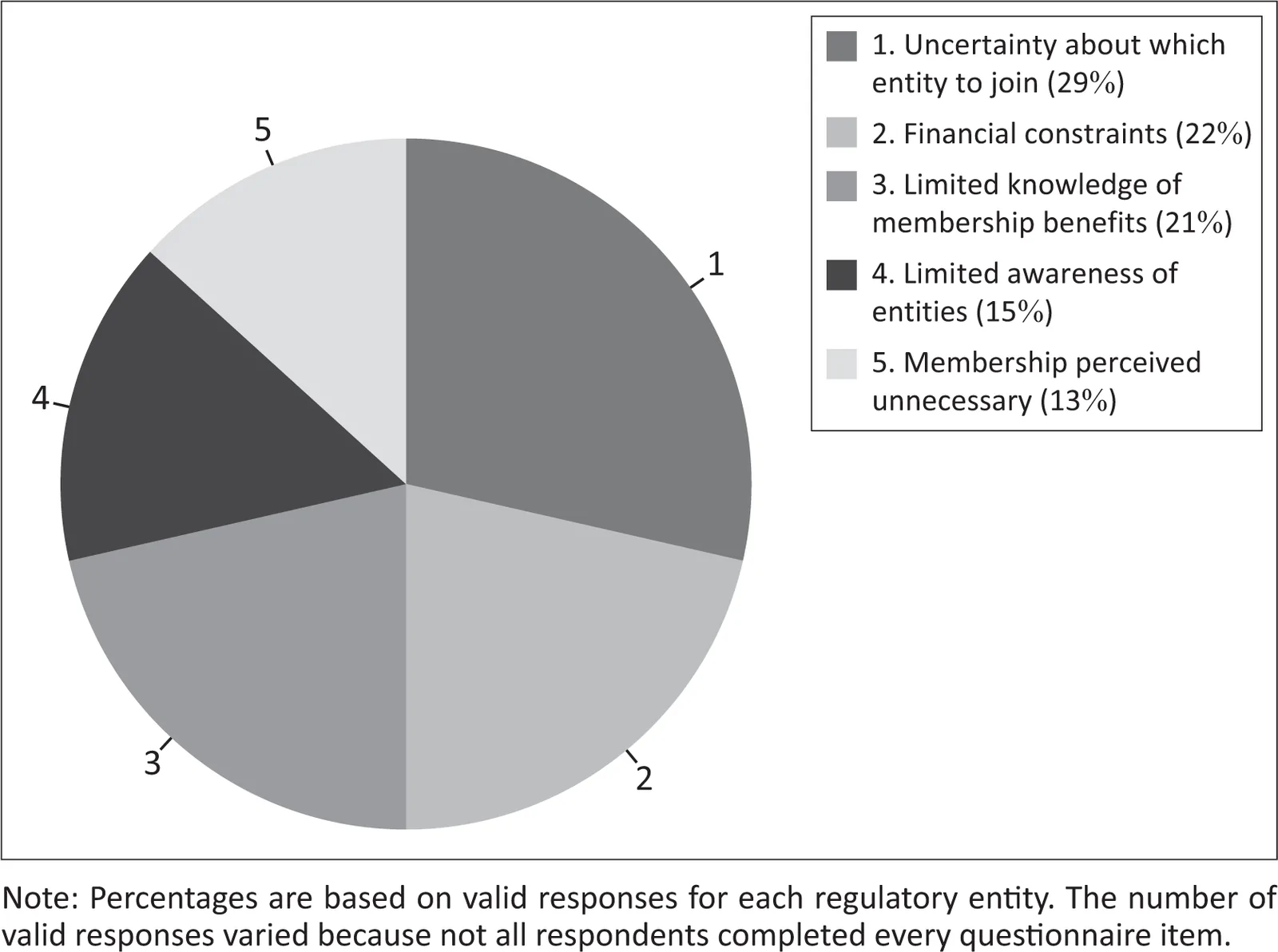
Challenges influencing non-membership percentage (*n* = 51).

#### Knowledge of guidelines and registration requirements

Table 4 summarises respondents’ knowledge of regulatory guidelines. South African Association of Health and Skincare Professionals demonstrated the highest level of guideline knowledge. Lower levels of guideline knowledge were recorded for AHPCSA, HPCSA, HCSBC, BHFSA and EOHCB as indicated in Table 4.

**TABLE 4:** Knowledge of guidelines and registration requirements.

Entity	%		Registration required	Highest %
	Guidelines: Not at all	Guidelines: Great extent		
AHPCSA	44.4	13.3	All somatologists	57.8
BHFSA	51.1	8.9	Employers	47.7
EOHCB	55.6	4.4	Employers	36.4
HCSBC	57.8	6.7	All somatologists	40.9
HPCSA	44.4	15.6	None required	33.3
SAAHSP	26.1	30.4	All somatologists	75.0

Note: Percentages are based on valid responses for each regulatory entity. The number of valid responses varied because not all respondents completed every questionnaire item.

HCSBC, National Bargaining Council for Hairdressing, Cosmetology, Beauty and Skincare Industry; AHPCSA, Allied Health Professions Council of South Africa; HPCSA, Health Professions Council of South Africa; BHFSA, Beauty Health and Skincare Employers Association; EOHCB, Employers’ Organisation for Hairdressing, Cosmetology and Beauty; SAAHSP, South African Association of Health and Skincare Professionals.

Table 4 also summarises respondents’ understanding of registration requirements. The response ‘all somatologists’ was most frequently selected for SAAHSP (75.0%). Allied Health Professions Council of South Africa followed at 57.8%, HCSBC at 40.9% and HPCSA at 50%. *Employers* were most often selected for BHFSA (47.7%) and EOHCB (36.4%). For HPCSA, 33.3% indicated that no registration was required.

#### Knowledge of continuing professional development

Figure 4 presents respondents’ knowledge of CPD requirements (*n* = 37). Fewer than 20% of respondents reported knowledge of CPD requirements.

**FIGURE 4:**
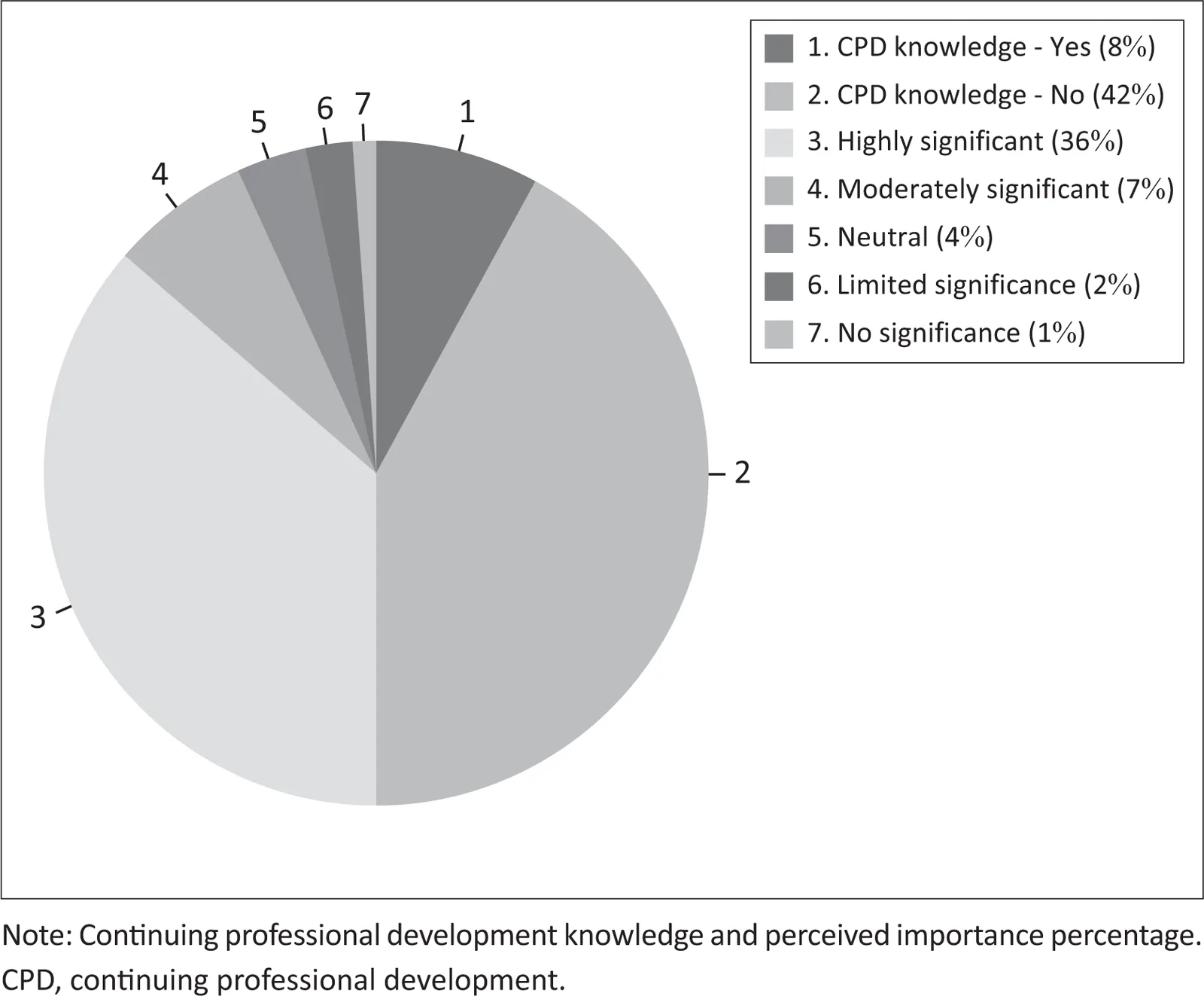
Knowledge of continuing professional development requirements (*n* = 37) and perceived significance of certification and professional development (*n* = 44).

Figure 4 also presents respondents’ perceptions of the significance of certification and professional development (*n* = 44). Most respondents (72.7%) regarded certification and professional development as highly significant. Although knowledge of CPD requirements was limited, the perceived value of certification and professional development was reported as high for most respondents.

## Discussion

Results indicate varying levels of awareness of regulatory entities; however, awareness does not equate to regulatory knowledge (Louw & Esterhuizen 2021; Van Niekerk 2020). Awareness denotes recognition of an entity, while knowledge requires understanding of mandate, authority and relevance to professional practice. Viewed through a professionalisation perspective, the findings suggest that regulatory literacy remains uneven. Although professional identity was not specifically assessed, the variability in regulatory knowledge may contribute to the development of professional identity, clearer scopes of practice and stronger accountability structures within the profession (Abbott 1988; Freidson 2001; Saks 2012). The present study focused on regulatory knowledge rather than broader constructs such as professional identity, confidence or regulatory readiness. Accordingly, interpretations of these broader concepts should be regarded as theoretical implications of the findings rather than as direct empirical observations. The discussion therefore moves beyond restating the results by considering how recognition, misunderstanding, and limited knowledge of guidelines shape professional identity, ethical accountability and readiness for future regulatory alignment within the South African somatology context.

### Knowledge of regulatory structures

Results demonstrate substantial variability in understanding of regulatory entities. Recognition was strongest for SAAHSP, which may reflect the association’s historical visibility, its professional networking role and its direct relevance to daily somatology practice (Ross et al. 2021; SAAHSP 2023). However, recognition did not consistently translate into an accurate understanding of governance authority. The attribution of statutory power to a voluntary association suggests that professional identity may be shaped by the most visible entity rather than by a clearly understood regulatory mandate. Although accountability was beyond the scope of the present study, such boundary confusion may influence the development of shared accountability and consistent interpretation of ethical and legal responsibilities (Cowie et al. 2020; Louw & Esterhuizen 2021).

Knowledge gaps increased in relation to AHPCSA, HPCSA, HCSBC, EOHCB and BHFSA. Respondents frequently misinterpreted statutory health entities as primary regulators of somatology or demonstrated limited clarity regarding labour-based organisational functions (HCSBC 2022). This finding extends existing literature by showing how fragmentation is reflected in practitioners’ knowledge patterns. While confidence and accountability were not examined in the present study, uncertainty regarding regulatory authority may nevertheless influence these aspects of professional practice. In the absence of a statutory regulator, knowledge formation appears to rely on informal industry exposure, resulting in uneven understanding and uncertain professional alignment.

### Recognition and familiarity

Familiarity with functional mandates was limited across most governance entities, aside from comparatively stronger familiarity with SAAHSP. Respondents frequently assigned statutory responsibilities to entities with labour, bargaining or professional representation functions. Recognition therefore occurred without a corresponding depth of conceptual understanding, reinforcing concerns regarding reduced regulatory literacy in emerging health professions (Ross et al. 2021). Limited guideline knowledge may also affect practice because somatologists who are unsure of entity functions may struggle to identify appropriate sources of guidance for ethical decisions, client safety concerns and advanced treatment responsibilities (SAQA 2022; WHO 2021).

### Membership patterns and challenges influencing affiliation

Membership activity was concentrated predominantly around SAAHSP, while minimal affiliation was recorded for other entities. Although perceptions of organisational visibility and relevance were not specifically assessed, these factors may have contributed to the observed pattern of membership. Challenges such as uncertainty about which entity to join, financial considerations and limited knowledge of membership benefits indicate that affiliation decisions remain discretionary and individually interpreted. From a professionalisation perspective, such uncertainty may slow the development of a collective professional identity because participation in governance structures is not yet perceived as a clear professional obligation (Louw & Esterhuizen 2021).

### Knowledge of guidelines and registration requirements

Results reveal substantial uncertainty regarding registration processes across occupational levels. Respondents often linked statutory entities to compulsory registration, despite the absence of statutory regulation for somatology. The incorrect allocation of employer or somatologist responsibilities to labour-focused entities further reflects governance misunderstanding. These findings may influence client safety and ethical accountability, as unclear registration expectations may result in inconsistent referral pathways, variable understanding of scope-related responsibility and uncertainty regarding where professional concerns should be directed (HCSBC 2022; Van Niekerk 2020).

### Knowledge of continuing professional development

Although organisational knowledge remains inconsistent, respondents recognised fundamental standards including hygiene, sterilisation, ethics, treatment safety and structured training. This indicates that practice-based professional values are present, even when structural regulatory knowledge is limited (WHO 2021). By contrast, knowledge of CPD requirements was minimal. The difference between valuing professional development and understanding formal CPD expectations suggests an important gap between professional intention and regulatory readiness. Strengthened CPD literacy may therefore support competence progression, responsiveness to advanced technologies and preparedness for future regulatory reform (Ross et al. 2021; SAQA 2022).

### Consolidated interpretation

A consolidated interpretation reveals that the central issue is not a complete absence of regulatory awareness, but an uneven movement from awareness to informed regulatory literacy. Strong recognition of SAAHSP, limited understanding of statutory health entities, uncertainty regarding labour-related entities, low familiarity with guidelines and minimal CPD literacy collectively indicate a profession in transition. These patterns may extend existing literature by suggesting how fragmented governance influences professional identity, decision-making and accountability at practice level. Regulatory knowledge is therefore a key developmental requirement for professionalisation, rather than a purely administrative concern.

### Recommendations and implications

Results emphasise the need for strengthened regulatory knowledge through coordinated information dissemination, guideline accessibility and structured orientation to regulatory entities. Training institutions could support this process by including dedicated curriculum content on regulatory entities, scope of practice, ethical accountability, CPD expectations and referral pathways. Existing entities could strengthen communication through concise comparison guides, accessible membership information, regular professional updates and orientation material for graduates and salon owners. These actions may reduce uncertainty, support informed decision-making and improve confidence in professional alignment within the Free State context, while also offering cautious evidence for broader national discussions.

## Conclusion

The findings reflect uneven regulatory knowledge among somatologists in the Free State. Recognition of SAAHSP was strong; however, knowledge relating to statutory councils, labour-aligned entities, guideline structures, registration requirements and CPD frameworks remained limited. Awareness rarely developed into informed comprehension, indicating a governance environment characterised by fragmentation and inconsistent dissemination of regulatory information. Knowledge appeared to develop through professional exposure rather than structured guidance, contributing to variability in interpretation and uncertainty when navigating regulatory obligations.

Respondents demonstrated a clear understanding of fundamental professional standards such as hygiene, ethics, client safety and procedural competence, and strongly valued continued professional development. However, limited familiarity with formal CPD requirements and entity-linked guidelines suggests the need for clearer communication, accessible regulatory information and structured knowledge pathways. Advancing somatology in South Africa will therefore depend on strengthening regulatory literacy to support safe practice, informed decision-making and unified professional identity.

### Study limitations and future directions

Strengthening regulatory knowledge could be supported through curriculum-integrated regulatory literacy in somatology training programmes, ensuring that new somatologists enter the industry with a clearer understanding of entities, guidelines, scope-related responsibilities and professional development expectations. Recommended curriculum components include the comparison of relevant entities, the interpretation of ethical and safety guidelines, CPD planning and the case-based application of regulatory decision-making. Regulatory literacy could be strengthened through structured incorporation of regulatory literacy into business management and professional practice modules. The inclusion of topics such as regulatory entities, ethical accountability, scope of practice, registration processes, CPD requirements and professional responsibilities may improve preparedness for industry participation and future statutory alignment. Practical activities such as case studies, entity comparisons, guest presentations by professional associations, and the interpretation of regulatory scenarios may further support an applied understanding of governance structures within the profession. More consistent communication from existing entities, including simplified guidance documents, webinars, graduate orientation resources and clear membership pathways, may further enhance understanding and support purposeful professional engagement.

The investigation was limited to the Free State province, which restricts the extent to which the findings can be generalised to the wider South African somatology industry. Convenience sampling further narrowed representativeness, while the electronic format of the questionnaire may have excluded somatologists with limited digital access. As the data relied on self-reported responses, the possibility of response bias, particularly in relation to professional conduct and regulatory engagement, cannot be excluded. In addition, the use of descriptive statistical procedures restricted the study to exploratory interpretation rather than predictive or inferential analysis. Despite these constraints, the methodological approach remained appropriate for the exploratory purpose of the enquiry.

Enhancing regulatory understanding offers a pathway to stronger professional credibility, improved client protection and sustainable industry development. The findings are specific to the Free State respondent group and should therefore be applied cautiously beyond the study context. Nevertheless, the patterns identified may inform wider professional discussions by highlighting the need for coherent communication, structured education and clearer direction regarding future regulatory alignment within the South African somatology industry.
